# Diallyl Trisulfide Suppresses Oxidative Stress-Induced Activation of Hepatic Stellate Cells through Production of Hydrogen Sulfide

**DOI:** 10.1155/2017/1406726

**Published:** 2017-02-20

**Authors:** Feng Zhang, Huanhuan Jin, Li Wu, Jiangjuan Shao, Xiaojing Zhu, Anping Chen, Shizhong Zheng

**Affiliations:** ^1^Jiangsu Key Laboratory for Pharmacology and Safety Evaluation of Chinese Materia Medica, Nanjing University of Chinese Medicine, Nanjing 210023, China; ^2^Jiangsu Key Laboratory of Therapeutic Material of Chinese Medicine, Nanjing University of Chinese Medicine, Nanjing 210023, China; ^3^State Key Laboratory Cultivation Base for TCM Quality and Efficacy, Nanjing University of Chinese Medicine, Nanjing 210023, China; ^4^Department of Pathology, School of Medicine, Saint Louis University, Saint Louis, MO 63104, USA

## Abstract

Accumulating data reveal that garlic has beneficial effects against chronic liver disease. We previously reported that diallyl trisulfide (DATS), the primary organosulfur compound in garlic, reduced fibrosis and attenuated oxidative stress in rat fibrotic liver. The present study was aimed at elucidating the underlying mechanisms. The primary rat hepatic stellate cells (HSCs) were cultured and stimulated with hydrogen peroxide (H_2_O_2_) for inducing HSC activation under oxidative stress. We examined the effects of DATS on the profibrogenic properties and oxidative stress in H_2_O_2_-treated HSCs. The results showed that DATS suppressed and reduced fibrotic marker expression in HSCs. DATS arrested cell cycle at G2/M checkpoint associated with downregulating cyclin B1 and cyclin-dependent kinase 1, induced caspase-dependent apoptosis, and reduced migration in HSCs. Moreover, intracellular levels of reactive oxygen species and lipid peroxide were decreased by DATS, but intracellular levels of glutathione were increased in HSCs. Furthermore, DATS significantly elevated hydrogen sulfide (H_2_S) levels within HSCs, but iodoacetamide (IAM) reduced H_2_S levels and significantly abrogated DATS production of H_2_S within HSCs. IAM also abolished all the inhibitory effects of DATS on the profibrogenic properties and oxidative stress in HSCs. Altogether, we demonstrated an H_2_S-associated mechanism underlying DATS inhibition of profibrogenic properties and alleviation of oxidative stress in HSCs. Modulation of H_2_S production may represent a therapeutic remedy for liver fibrosis.

## 1. Introduction

Hepatic fibrosis represents an overactive wound healing process secondary to a variety of chronic liver injuries. During fibrogenesis, excessive connective tissue accumulates in the liver and results in the distortion of hepatic architecture [1]. Chronic liver injury activates and transforms quiescent hepatic stellate cells (HSCs) from vitamin A-storing pericytes to myofibroblast-like cells. Once activated, HSCs become profibrogenic through acquiring proliferative, contractile, and proinflammatory properties as the primary cellular source of extracellular matrix (ECM) components in the liver [2]. Clinical and basic data suggest that oxidative stress critically participates in the progression of fibrosis and acts as mediators of molecular and cellular events implicated in liver fibrosis. Generation of reactive oxygen species (ROS) plays an important role in HSC activation and initiation of hepatic fibrogenesis [3]. Culture of HSCs treated with oxidative stress-related molecules can mimic the HSC activation caused by oxidative stress in liver fibrosis [4]. This cellular model is commonly established for investigating the mechanisms of HSC activation and evaluating the effects of antifibrotic candidates. However, currently there are few breakthroughs in the therapeutic intervention of hepatic fibrosis. Identification of antifibrogenic agents that are innocuous is urgently needed.

Garlic has been recognized for prevention and treatment of various diseases by many different cultures throughout history. Recent studies support the effects of garlic and its functional ingredients in a wide range of applications, including anticancer, antithrombotic, antiatherosclerotic, antidiabetic, and antioxidant properties [5]. It is characterized that diallyl trisulfide (DATS), a major organosulfur compound in garlic, is responsible for the pharmacological efficacy of garlic [6]. We previously demonstrated that DATS reduced carbon tetrachloride (CCl_4_)-caused liver injury and fibrogenesis in rats, which was associated with inhibition of HSC activation and attenuation of hepatic oxidative stress [7]. However, the molecular mechanisms underlying DATS's antifibrotic activity are not fully understood. Interestingly, emerging data indicate that drugs that target hydrogen sulfide (H_2_S) or generate safe levels of H_2_S in vivo may be therapeutic options for chronic liver diseases [8]. It is given that DATS is an exogenous donor of H_2_S and the liver has a high hepatic capacity for H_2_S metabolism [9]. We hypothesized that DATS exerted its antifibrotic effects associated with generation of H_2_S. Experiments were performed to verify this hypothesis.

## 2. Materials and Methods

### 2.1. Reagents and Antibodies

The following compounds were used in this study: DATS (purity > 97%; Shenzhen Minn Bolin Biotechnology Co., Ltd., Shenzhen, China), iodoacetamide (IAM; Nanjing Dingguo Changsheng Biotechnology Co., Ltd., Nanjing, China), and mitomycin (Sigma, St Louis, MO, USA). They were dissolved in dimethylsulfoxide (DMSO) for experiments. DMSO at a concentration of 0.02% (w/v) was set as a vehicle control throughout the studies. Analytical grade 30% hydrogen peroxide (H_2_O_2_; Sinopharm Chemical Reagent Co., Ltd., Shanghai, China) was diluted with deionized water to indicated concentrations for experiments. The following primary antibodies were used in this study: *α*-SMA, *α*1(I) procollagen, and fibronectin (Epitomics, San Francisco, CA, USA); TGF-*β*RI, TGF-*β*RII, PDGF-*β*R, EGF-R, and Bcl-2 (Santa Cruz Biotechnology, Santa Cruz, CA, USA); cyclin A, cyclin B1, CDK1, CDK2, Bax, pro-caspase-9, cleaved-caspase-9, pro-caspase-8, cleaved-caspase-8, pro-caspase-7, cleaved-caspase-7, pro-caspase-3, cleaved-caspase-3, full-length PARP-1, cleaved-PARP-1, and *β*-Actin (Cell Signaling Technology, Danvers, MA, USA).

### 2.2. Cell Culture

Primary rat HSCs were obtained from Jiangyin CHI Scientific, Inc. (Wuxi, China). HSCs were cultured in Dulbecco's modified eagle medium (DMEM; Invitrogen, Grand Island, NY, USA) with 10% fetal bovine serum (FBS; Wisent Biotechnology Co., Ltd., Nanjing, China), 1% antibiotics, and grown in a 5% CO_2_ humidified atmosphere at 37°C. Cell morphology was assessed under an inverted microscope (Leica, Germany).

### 2.3. Cytotoxicity Assay

HSCs were seeded in 24-well plates and cultured for 24 h. HSCs were then treated with DMSO or DATS at indicated concentrations for 24 h. Lactate dehydrogenase (LDH) activity in culture medium was determined using LDH cytotoxicity assay kits (Nanjing Jiancheng Bioengineering Institute, Nanjing, China) according to the protocol. Results were from triplicate experiments.

### 2.4. Cell Viability Assay

HSCs were seeded in 96-well plates and cultured for 24 h. HSCs were then treated with H_2_O_2_ and/or DATS at indicated concentrations for 24 h. The medium was replaced with 100 *μ*L phosphate buffered saline (PBS) containing 0.5 mg/mL 3-(4,5-dimethylthiazol-2-yl)-2,5-diphenyl tetrazolium bromide (MTT) and cells were maintained at 37°C for 4 h. Then, the crystals were dissolved with 200 *μ*L DMSO. The spectrophotometric absorbance was measured with a SPECTRAmax™ microplate spectrophotometer (Molecular Devices, Sunnyvale, CA, USA) at 490 nm. Results were from triplicate experiments.

### 2.5. Enzyme-Linked Immunosorbent Assay (ELISA) for H_2_S

HSCs were seeded in 6-well plates and cultured for 24 h. HSCs were then treated with DATS and/or IAM at indicated concentrations for 24 h. Cells were then lysated with RIPA buffer. Intracellular H_2_S concentrations were determined using an ELISA kit purchased from Nanjing Jiancheng Bioengineering Institute (Nanjing, China) according to the protocol. Experiments were performed in triplicate.

### 2.6. Cell Cycle Analyses

HSCs were seeded in 6-well plates and cultured for 24 h. HSCs were then treated with various reagents at indicated concentrations for 24 h and harvested and fixed. Cell cycle was analyzed using cellular DNA flow cytometric kits (Nanjing KeyGen Biotech Co., Ltd., Nanjing, China) according to the protocol. Percentages of cells within cell cycle compartments were determined by flow cytometry (FACSCalibur; BD, Franklin Lakes, NJ, USA). Results were from triplicate experiments.

### 2.7. Apoptosis Analyses

HSCs were seeded in 6-well plates and cultured for 24 h. HSCs were then treated with various reagents at indicated concentrations for 24 h. Morphology of apoptotic HSCs was evaluated using Hoechst staining kits (Nanjing KeyGen Biotechnology Co., Ltd., Nanjing, China) according to the protocol. Photographs were taken under a fluorescence microscope (Nikon, Tokyo, Japan). In certain experiments, the apoptotic rates were determined by flow cytometry using Annexin V-FITC apoptosis assay kits (Nanjing KeyGen Biotech Co., Ltd., Nanjing, China) according to the protocol. Apoptotic cells were defined as the cells situated in the right two quadrants of each plot and the percentages were determined by flow cytometry (FACSCalibur; BD, Franklin Lakes, NJ, USA). Results were from triplicate experiments.

### 2.8. Wound Healing Assay

HSCs were seeded in 6-well plates and cultured in DMEM with 10% FBS. Once the cells attached properly, they were treated with mitomycin at 4 *μ*g/mL for 3 h. One linear wound was scraped in each well with a sterile pipette tip, and cells were washed with PBS to remove the unattached cells. Then cells were treated with various reagents at indicated concentrations. Images were taken at 12 h after wound induction at the same field under an inverted microscope (Leica, Germany). Results were from triplicate experiments.

### 2.9. Boyden Chamber Assay

HSCs were seeded to the upper wells of polycarbonate membrane transwell inserts (8 *μ*m pore-size; Corning, USA) and cultured in DMEM with 10% FBS, and meanwhile they were treated with various reagents at indicated concentrations. The lower chambers were filled with complete medium and H_2_O_2_ at 5 *μ*M. After 12 h incubation, the polycarbonated filter was removed and the migrated cells on the lower surface were stained with crystal violet. The number of migrated cells at five random fields for each well was counted and normalized to control. Results were from triplicate experiments.

### 2.10. Measurement of ROS

Analysis of intracellular ROS was determined using dichlorofluorescein diacetate (DCFH-DA) probe (Shanghai Genmed Co., Ltd., Shanghai, China). DCFH-DA is an oxidation-sensitive nonfluorescent precursor dye that can be oxidized by H_2_O_2_, other ROS, and low molecule weight peroxides to fluorescent DCFH. HSCs were seeded in 6-well plates and cultured for 24 h. HSCs were then treated with various reagents at indicated concentrations for 24 h. In certain experiments, cells were washed with PBS and incubated with DCFH-DA solutions (10 *μ*M) for 20 min. Micrographs were taken under a fluorescence microscope (Nikon, Tokyo, Japan). The excitation and emission wavelengths were set at 488 and 525 nm, respectively. In certain experiments, cells were detached by trypsinization and suspended in DCFH-DA solution (10 *μ*M) for 20 min. The intensity of cellular fluorescence intensity was determined by flow cytometry (FACSCalibur; BD, Franklin Lakes, NJ, USA). Results were from triplicate experiments.

### 2.11. Measurement of Lipid Peroxide (LPO)

HSCs were seeded in 6-well plates and cultured for 24 h. HSCs were then treated with various reagents at indicated concentrations for 24 h and were lysated with RIPA buffer. Intracellular LPO levels were determined using kits purchased from Nanjing Jiancheng Bioengineering Institute (Nanjing, China) according to the protocol. Experiments were performed in triplicate.

### 2.12. Measurement of Glutathione (GSH)

HSCs were seeded in 6-well plates and cultured for 24 h. HSCs were then treated with various reagents at indicated concentrations for 24 h and were lysated with RIPA buffer. Intracellular GSH levels were determined using kits purchased from Nanjing Jiancheng Bioengineering Institute (Nanjing, China) according to the protocol. Experiments were performed in triplicate.

### 2.13. Western Blot Analyses

HSCs were treated with various reagents at indicated concentrations for 24 h. Whole cell protein preparation, detection, and band visualization were performed as we previously described [7]. *β*-Actin was used as an invariant control for equal loading of total proteins. Representative blots were from three independent experiments.

### 2.14. Statistical Analyses

Data were presented as mean ± SD and analyzed using SPSS 16.0 software. Histograms were created using GraphPad Prism 5 software (San Diego, CA, USA). The significance of difference was determined by one-way ANOVA with the post hoc Dunnett's test. Values of *P* < 0.05 were considered to be statistically significant.

## 3. Results

### 3.1. DATS Inhibits Viability and Reduces Fibrotic Protein Expression Associated with H_2_S in HSCs

**Figure 1:**
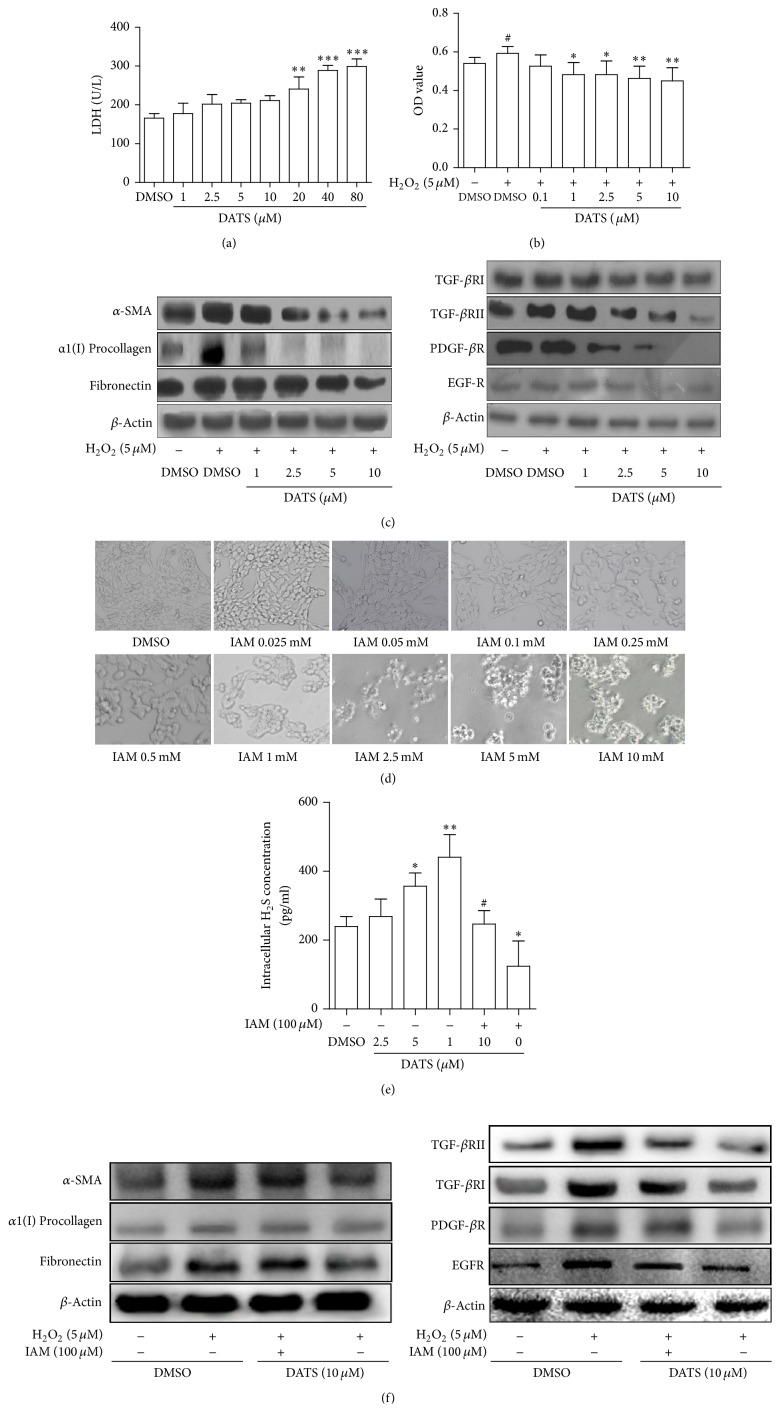
DATS inhibits viability and reduces fibrotic protein expression associated with H_2_S in HSCs. (a) LDH release assay. Significance: ^*∗∗*^*P* < 0.01 versus DMSO and ^*∗∗∗*^*P* < 0.001 versus DMSO. (b) MTT assay. Significance: ^#^*P* < 0.05 versus DMSO, ^*∗*^*P* < 0.05 versus DMSO + H_2_O_2_, and ^*∗∗*^*P* < 0.01 versus DMSO + H_2_O_2_. (c) Western blot analyses of profibrotic marker proteins. (d) Morphology evaluation with light microscope (200x magnification). (e) ELISA for H_2_S concentration. Significance: ^*∗*^*P* < 0.05 versus DMSO, ^*∗∗*^*P* < 0.01 versus DMSO, and ^#^*P* < 0.05 versus DATS 10 *μ*M. (f) Western blot analyses of profibrotic marker proteins.

We initially evaluated potential cytotoxicity of DATS on HSCs using LDH assay, showing that DATS at 20 *μ*M or higher concentrations had significant cytotoxic effects on HSCs (Figure 1(a)). We thus investigated DATS effects at nontoxic doses subsequently. We herein used H_2_O_2_ to stimulate HSC activation mimicking oxidative stress, because H_2_O_2_ is the most stable ROS and diffuses readily in and out of cells [10]. DATS at doses of 0.1–10 *μ*M inhibited cell viability concentration-dependently in H_2_O_2_-stimulated HSCs (Figure 1(b)). Moreover, DATS reduced the expression of fibrotic marker proteins *α*-smooth muscle actin (*α*-SMA), *α*1(I) procollagen, and fibronectin and downregulated the type I and II receptors for transforming growth factor-*β* (TGF-*β*RI and TGF-*β*RII), platelet-derived growth factor-*β* receptor (PDGF-*β*R), and epidermal growth factor receptor (EGF-R) in H_2_O_2_-stimulated HSCs (Figure 1(c)). We hypothesized that DATS exerted its effects associated with production of H_2_S. The compound IAM has been demonstrated to block both the exofacial and intracellular thiols resulting in elimination of garlic-induced H_2_S production [11]. Here, IAM at concentration range within 100 *μ*M did not cause morphological alterations in HSCs, and accordingly we selected 100 *μ*M IAM for subsequent experiments (Figure 1(d)). We found that DATS concentration-dependently increased the H_2_S content in HSCs and that IAM at 100 *μ*M significantly abrogated DATS elevation of H_2_S production and reduced the base level of H_2_S in HSCs (Figure 1(e)), highlighting IAM as a potent blocker of DATS generation of H_2_S. Furthermore, IAM effectively rescued DATS downregulation of fibrotic marker molecules in H_2_O_2_-stimulated HSCs (Figure 1(f)). Taken together, these data suggested that H_2_S was involved in DATS suppression of the expression of fibrotic proteins in activated HSCs.

### 3.2. DATS Induces Cell Cycle Arrest Associated with H_2_S in HSCs

**Figure 2:**
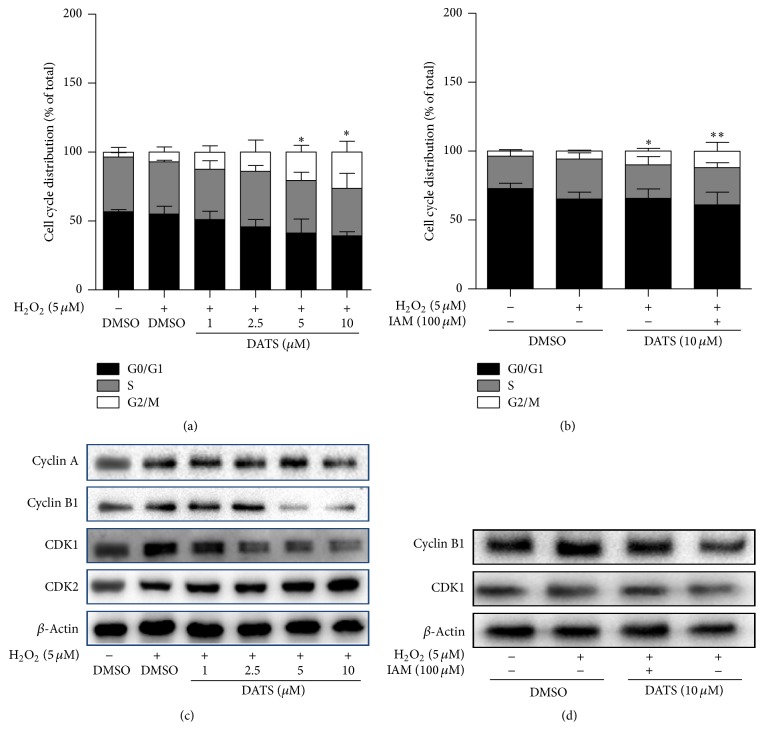
DATS induces cell cycle arrest associated with H_2_S in HSCs. (a, b) Percentages of cell cycle distributions were determined by flow cytometry. Significance: ^*∗*^*P* < 0.05 versus DMSO + H_2_O_2_ and ^*∗∗*^*P* < 0.01 versus DMSO + H_2_O_2_. (c, d) Western blot analyses of cell cycle regulatory proteins.

We next examined DATS effects on cell fate of HSCs. DATS arrested cell cycle at G2/M checkpoint in H_2_O_2_-stimulated HSCs (Figure 2(a)). Addition of IAM abrogated DATS-induced G2/M arrest (Figure 2(b)). Cyclin A, cyclin B1, cyclin-dependent kinase (CDK) 1, and CDK2 are four critical regulators at G2/M checkpoint responsible for driving cells into the mitosis process [12]. DATS decreased the expression of cyclin B1 and CDK1, while cyclin A and CDK2 were not apparently affected by DATS (Figure 2(c)). Moreover, DATS inhibition of cyclin B1 and CDK1 was abolished by IAM (Figure 2(d)). These data collectively indicated that H_2_S was involved in DATS modulation of cell cycle regulators and G2/M checkpoint arrest in HSCs.

### 3.3. DATS Stimulates Apoptosis Associated with H_2_S in HSCs

**Figure 3:**
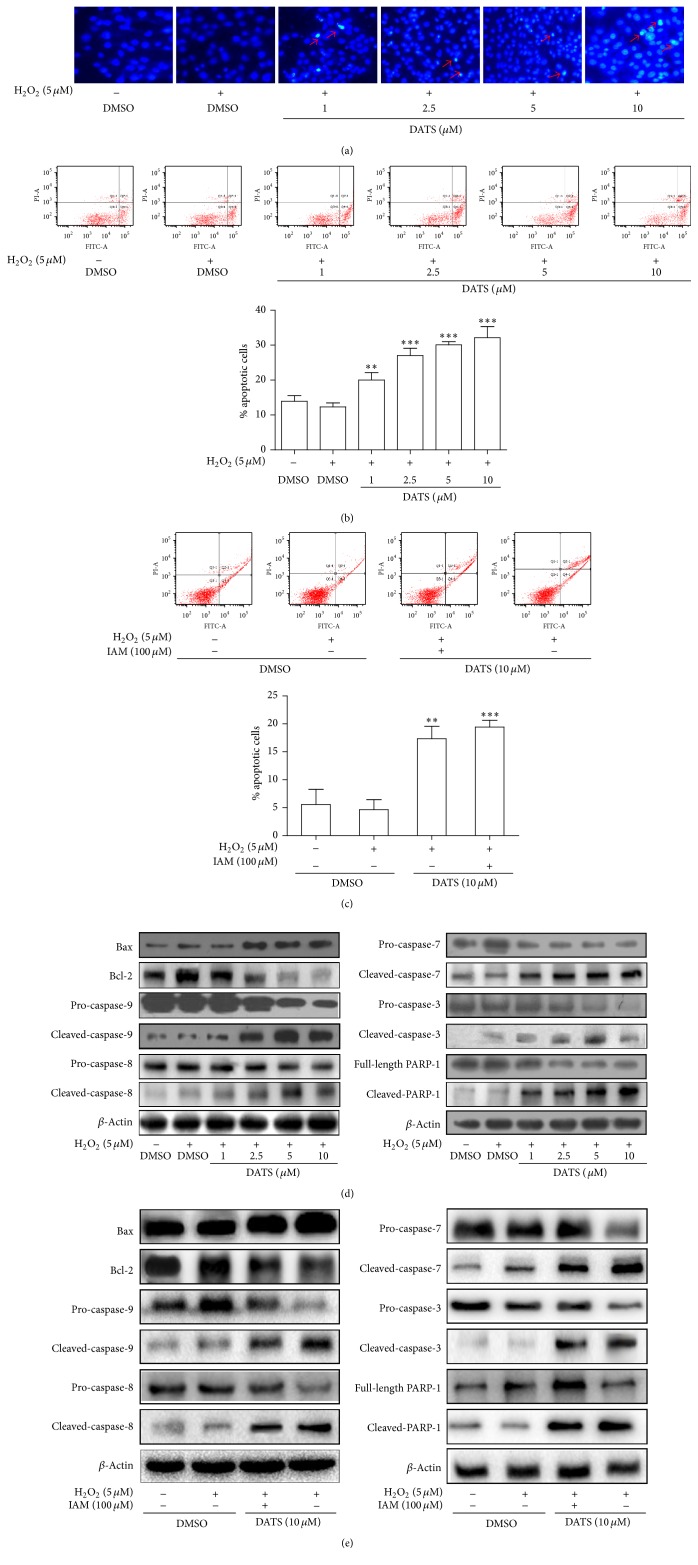
DATS stimulates apoptosis associated with H_2_S in HSCs. (a) Hoechst fluorescence staining for morphology of apoptotic HSCs under a fluorescence microscope (200x magnification). (b, c) Flow cytometric analyses of apoptotic rates. Significance: ^*∗∗*^*P* < 0.01 versus DMSO + H_2_O_2_ and ^*∗∗∗*^*P* < 0.001 versus DMSO + H_2_O_2_. (d, e) Western blot analyses of apoptosis regulatory proteins.

We subsequently examined DATS effects on HSC apoptosis. HSCs treated with DATS exhibited significant DNA condensation and fragmentation with brilliant blue staining (Figure 3(a)), and DATS increased apoptotic rates concentration-dependently in H_2_O_2_-treated HSCs (Figure 3(b)). However, the increase in apoptotic rate by DATS was abolished by IAM (Figure 3(c)). Furthermore, the antiapoptotic protein Bcl-2 was diminished by DATS; the proapoptotic molecule Bax was increased by DATS (Figure 3(d)). DATS also activated the caspase cascade in H_2_O_2_-stimulated HSCs, because the cleaved forms of caspase-9, caspase-8, caspase-7, and caspase-3, and PARP-1 were all increased by DATS (Figure 3(d)). However, DATS effects on these molecules were abrogated by IAM (Figure 3(e)). Altogether, these findings indicated that H_2_S was involved in DATS induction of caspase-mediated apoptosis in HSCs.

### 3.4. DATS Inhibits Migration Associated with H_2_S in HSCs

**Figure 4:**
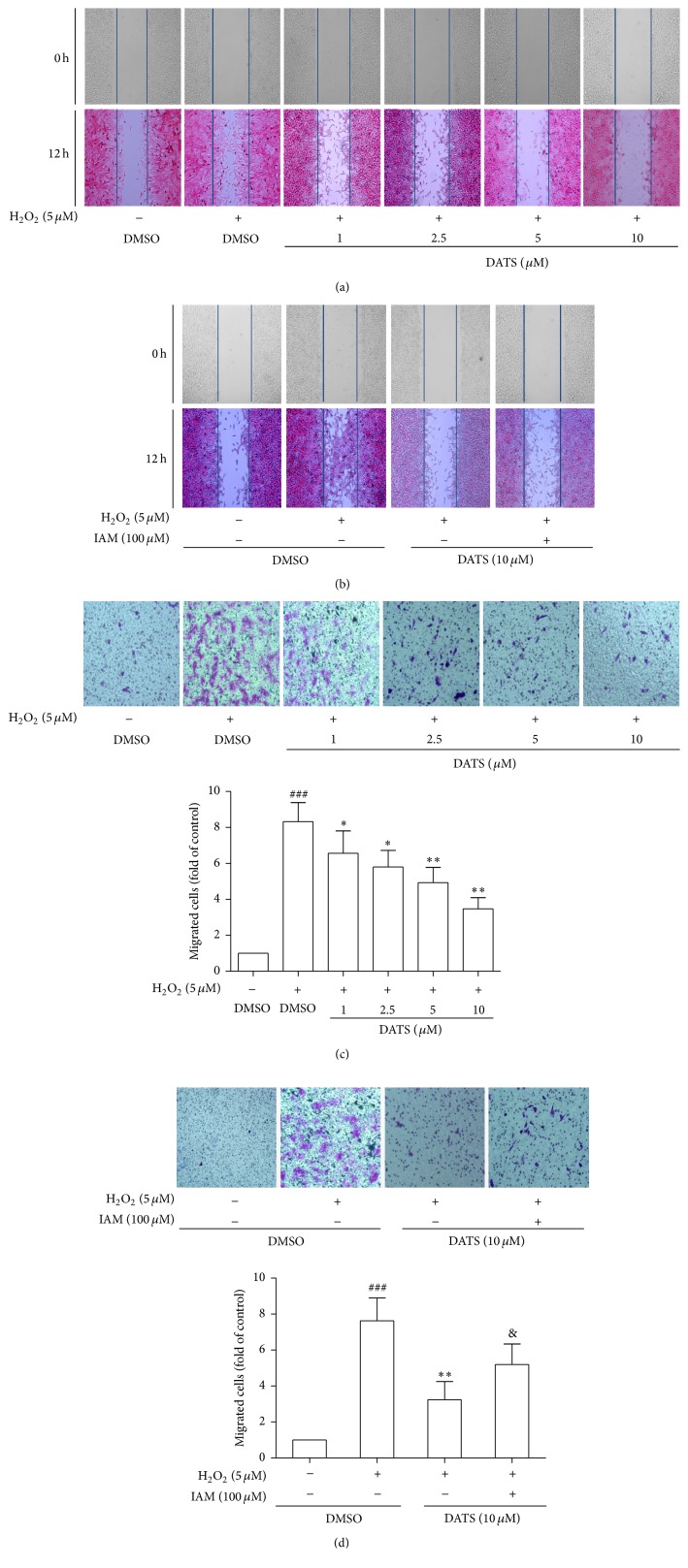
DATS inhibits migration associated with H_2_S in HSCs. (a, b) Wound healing assay (50x magnification). (c, d) Boyden chamber assay (50x magnification). Significance: ^###^*P* < 0.001 versus DMSO, ^*∗*^*P* < 0.05 versus DMSO + H_2_O_2_, ^*∗∗*^*P* < 0.01 versus DMSO + H_2_O_2_, and ^&^*P* < 0.05 versus DATS + H_2_O_2_.

We next examined DATS effects on migration in HSCs. DATS inhibited the lateral migration of H_2_O_2_-activated HSCs (Figure 4(a)). However, IAM abrogated DATS's inhibitory effects on HSC lateral migration (Figure 4(b)). To confirm the results, we performed transwell migration assay to evaluate the vertical migration of HSCs, showing that H_2_O_2_-stimulated vertical migration was suppressed by DATS (Figure 4(c)). Similarly, DATS effects were also significantly compensated by IAM (Figure 4(d)). Collectively, these data indicated that DATS inhibition of cell migration was associated with H_2_S in HSCs.

### 3.5. DATS Alleviates Oxidative Stress Associated with H_2_S in HSCs

**Figure 5:**
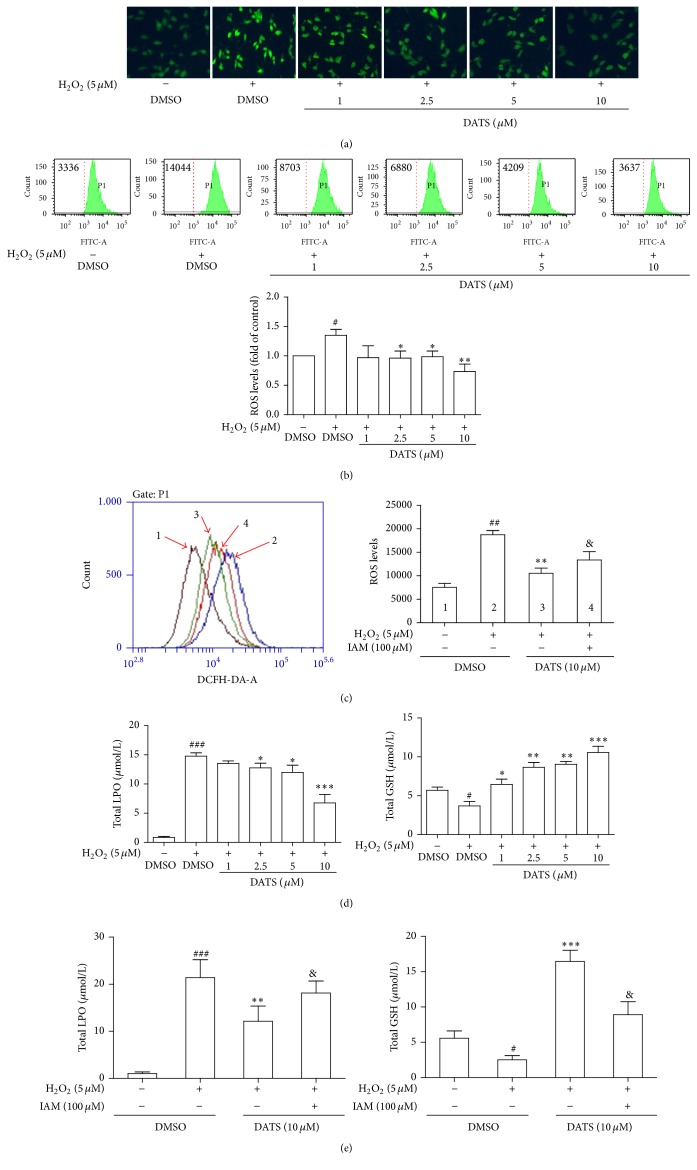
DATS alleviates oxidative stress associated with H_2_S in HSCs. (a) Analyses of intracellular ROS using a fluorescence microscope (100x magnification). (b, c) Analyses of intracellular ROS using flow cytometry with quantification. Significance: ^#^*P* < 0.05 versus DMSO, ^##^*P* < 0.01 versus DMSO, ^*∗*^*P* < 0.05 versus DMSO + H_2_O_2_, ^*∗∗*^*P* < 0.01 versus DMSO + H_2_O_2_, and ^&^*P* < 0.05 versus DATS + H_2_O_2_. (d, e) Analyses of intracellular LPO and GSH. Significance: ^#^*P* < 0.05 versus DMSO, ^###^*P* < 0.001 versus DMSO, ^*∗*^*P* < 0.05 versus DMSO + H_2_O_2_, ^*∗∗*^*P* < 0.01 versus DMSO + H_2_O_2_, ^*∗∗∗*^*P* < 0.001 versus DMSO + H_2_O_2_, and ^&^*P* < 0.05 versus DATS + H_2_O_2_.

We finally determined DATS effects on oxidative stress in HSCs. Fluorescence microscope analyses and flow cytometry assessments consistently demonstrated that H_2_O_2_-treated HSCs had significantly high intracellular levels of ROS, but DATS decreased ROS production (Figures 5(a) and 5(b)). However, IAM significantly rescued DATS-caused reduction in ROS levels in H_2_O_2_-stimulated HSCs (Figure 5(c)). Furthermore, DATS decreased the intracellular levels of LPO and increased the intracellular levels of GSH in H_2_O_2_-stimulated HSCs (Figure 5(d)). However, DATS inhibition of LPO and elevation of GSH were significantly compensated by IAM (Figure 5(e)). Altogether, these results suggested that DATS attenuated oxidative stress in HSCs, which was relevant to H_2_S.

## 4. Discussion

Many studies demonstrated that garlic extracts elicited therapeutic effects against liver fibrosis. Garlic extracts restored liver histology and accelerated ECM degradation, resulting in regression of liver fibrosis in CCl_4_-intoxicated rats [13]. Administration of aqueous garlic extracts alleviated hepatic oxidative injury and reduced collagen content in bile duct ligation-induced fibrosis in rats [14]. However, these investigations did not identify the specific functional ingredients responsible for the antifibrotic efficacy of garlic. Our previous data showed that DATS attenuated collagen deposition, inhibited HSC activation, and ameliorated oxidative stress in rat fibrotic liver, strengthening the therapeutic value of garlic for liver fibrosis [7]. Elucidation of the underlying mechanisms would be essentially important for developing DATS as a promising antifibrotic candidate.

Our current experiments could provide consistent support for the prior in vivo data. We found that DATS inhibited HSC activation in culture, which confirmed that HSCs could be target cells for DATS, because DATS was able to inhibit nearly all aspects of HSC activation. To explore the underlying mechanisms, we concentrated on the potential biotransformation of DATS. A 2007 study firstly described the real-time kinetics of H_2_S production of DATS within cells, showing that DATS with allyl moieties and three sulfur atoms reacted with exofacial membrane thiols and cross the cell membrane to react with GSH to produce H_2_S, which was a thiol-dependent manner for DATS to produce H_2_S [11]. Thereafter, increasing evidence reveals that the beneficial effects of garlic-derived organic polysulfides are mediated by production of H_2_S. Herein, we thus strongly speculated that H_2_S contributed to DATS inhibition of HSC activation. As expected, DATS could significantly increase H_2_S levels concentration-dependently in HSCs in current study. Moreover, we reasoned that IAM could be a proper blocker of DATS production of H_2_S, because their thiol-dependent mechanisms of action were exactly matching. Consistently, we here observed that IAM not only abolished DATS elevation of H_2_S production, but also reduced basal H_2_S synthesis in HSCs. Accordingly, we used IAM to testify the role of H_2_S in DATS inhibition of HSC activation properties. The obtained results clearly demonstrated that this H_2_S manipulation significantly weakened DATS effects. We identified the H_2_S as a molecular link implicated in DATS's antifibrotic activity. Actually, the role of H_2_S in hepatology has been studied increasingly during recent years. Malfunction of hepatic H_2_S metabolism may be involved in many chronic liver diseases, including hepatic fibrosis and cirrhosis [15]. Exogenous H_2_S could reduce hepatotoxicity, liver cirrhosis, and portal hypertension through multiple functions including antioxidation, anti-inflammation, cytoprotection, and antifibrosis [16]. Our current data were consistent with these observations and provided evidence that DATS could be transformed to be H_2_S that mediated the potent hepatoprotective and antifibrotic effects in HSCs.

We demonstrated that H_2_S was involved in DATS effects on many aspects of HSC activation, including fibrotic marker expression, cell cycle, apoptosis, migration, and the status of oxidative stress. Consistent with our data, recent investigations showed that sodium hydrosulfide, a well-known inorganic H_2_S-releasing molecule, suppressed proliferation and induced G1 phase cell cycle arrest in HSCs [17] and attenuated collagen expression in rats with CCl_4_-induced hepatic fibrosis [18]. These findings collectively suggested H_2_S as a signaling molecule regulating the pathophysiology of HSCs. Actually, H_2_S promotes a number of cellular signals that regulate metabolism, proliferation, and apoptosis in various types of cells. For example, exogenous treatment with H_2_S led to growth inhibition with cyclin D1 downregulation in rat smooth muscle cells [19] and stimulated apoptosis by downregulation of Bcl-2 and Bcl-xL in portal vein smooth muscle cells [20]. These observations were recaptured in HSCs by our present study and potentiated the role of H_2_S as a gaseous signaling molecule with a DNA damaging function. Interestingly, there was evidence that administration of sodium hydrosulfide significantly inhibited hepatocyte apoptosis in hepatic ischemia reperfusion-induced injury in rats, suggesting a hepatoprotective role for H_2_S in vivo [21]. However, it should be noted that this study examined apoptosis molecules in whole liver tissues rather than in single type of cells. This may raise a possibility that the proapoptotic effects of H_2_S on HSCs could be masked due to the overwhelming amount of hepatocytes in the liver. It could also be assumed that the different effects of H_2_S on apoptosis were cell-specific and disease-specific. Furthermore, we found that DATS inhibited HSC migration associated with H_2_S. Consistently, recent studies showed that DATS suppressed migration and invasion in human colon cancer cells [22] and breast cancer cells [23]. Another important issue is that increasing evidence elucidates the fundamental role of HSC in liver immunology, because HSCs represents a versatile source of many soluble immunological active factors and may act as an antigen presenting cell [24]. Understanding the role of HSCs as central regulators of liver immunology may lead to novel therapeutic strategies for chronic liver diseases. Interestingly, H_2_S has multiple and complex cellular signaling roles in relation to immunological modulation under certain pathophysiological conditions [25]. However, studies addressing how H_2_S regulates HSC immunology during hepatic fibrosis are not seen, which could be an attractive field in hepatology.

We herein evaluated DATS effects in an oxidative stress-induced HSC activation model, because signs of oxidative stress are concomitant or precede HSC activation and collagen deposition [3]. We also suggested a role for H_2_S in regulation of oxidative stress in HSCs. H_2_S could increase intracellular GSH concentrations and suppress oxidative stress in mitochondria [26]. H_2_S could also protect murine liver against ischemia reperfusion injury through upregulation of intracellular antioxidant pathways [27]. The antioxidant effects of H_2_S have recently been examined through use of H_2_S-releasing derivative ACS67, which was found to inhibit ROS formation and NADH oxidation in endothelial cells [28]. In current study, H_2_S also contributed to DATS attenuation of oxidative stress in HSCs. However, whether H_2_S exerted its antioxidant effects by virtue of its undoubted reducing activity or by its activation of endogenous defense systems merits further investigation. Furthermore, it is necessary to use an additional HSC line and another blocker of H_2_S generation to make the results more credible. There is also a need to carry out in vivo studies using certain H_2_S generation blocker to validate the H_2_S-dependent mechanism underlying the antifibrotic effects of DATS, which is ongoing in our laboratory.

In summary, DATS inhibited the profibrogenic properties and alleviated oxidative stress in HSCs. These effects were associated with production of H_2_S within cells. Our data elucidated, at least partially, the mechanisms underlying DATS's antifibrotic activity and indicated a therapeutic role of targeting H_2_S for treatment of liver fibrosis.
